# Enterovirus 71-induced autophagy increases viral replication and pathogenesis in a suckling mouse model

**DOI:** 10.1186/s12929-014-0080-4

**Published:** 2014-08-20

**Authors:** Ying-Ray Lee, Po-Shun Wang, Jen-Ren Wang, Hsiao-Sheng Liu

**Affiliations:** 1 Department of Medical Research, Chiayi Christian Hospital, Chiayi, Taiwan; 2 Department of Microbiology and Immunology, College of Medicine, National Cheng Kung University, Tainan, Taiwan; 3 Department of Medical Laboratory Science and Biotechnology, College of Medicine, National Cheng Kung University, Tainan, Taiwan; 4 Center of Infectious Disease and Signaling Research, College of Medicine, National Cheng Kung University, Tainan, Taiwan

**Keywords:** EV71, Autophagy, Amphisome, Suckling mice

## Abstract

**Background:**

We previously reported that Enterovirus 71 (EV71) infection activates autophagy, which promotes viral replication both *in vitro* and *in vivo*. In the present study we further investigated whether EV71 infection of neuronal SK-N-SH cells induces an autophagic flux. Furthermore, the effects of autophagy on EV71-related pathogenesis and viral load were evaluated after intracranial inoculation of mouse-adapted EV71 (MP4 strain) into 6-day-old ICR suckling mice.

**Results:**

We demonstrated that in EV71-infected SK-N-SH cells, EV71 structural protein VP1 and nonstructural protein 2C co-localized with LC3 and mannose-6-phosphate receptor (MPR, endosome marker) proteins by immunofluorescence staining, indicating amphisome formation. Together with amphisome formation, EV71 induced an autophagic flux, which could be blocked by NH_4_Cl (inhibitor of acidification) and vinblastine (inhibitor of fusion), as demonstrated by Western blotting. Suckling mice intracranially inoculated with EV71 showed EV71 VP1 protein expression (representing EV71 infection) in the cerebellum, medulla, and pons by immunohistochemical staining. Accompanied with these infected brain tissues, increased expression of LC3-II protein as well as formation of LC3 aggregates, autophagosomes and amphisomes were detected. Amphisome formation, which was confirmed by colocalization of EV71-VP1 protein or LC3 puncta and the endosome marker protein MPR. Thus, EV71-infected suckling mice (similar to EV71-infected SK-N-SH cells) also show an autophagic flux. The physiopathological parameters of EV71-MP4 infected mice, including body weight loss, disease symptoms, and mortality were increased compared to those of the uninfected mice. We further blocked EV71-induced autophagy with the inhibitor 3-methyladenine (3-MA), which attenuated the disease symptoms and decreased the viral load in the brain tissues of the infected mice.

**Conclusions:**

In this study, we reveal that EV71 infection of suckling mice induces an amphisome formation accompanied with the autophagic flux in the brain tissues. Autophagy induced by EV71 promotes viral replication and EV71-related pathogenesis.

## Background

EV71 is a non-enveloped positive-sense single-stranded RNA virus belonging to the *Enterovirus* genus. EV71 was first isolated from an infant suffering from aseptic meningitis in California in 1969 [1]. The first EV71 outbreak was reported in 1975, and epidemics of EV71 infection have been reported since the late 1990s in Asia-Pacific regions [2]–[6]. EV71 infection mainly causes hand, foot and mouth disease (HFMD), and most fatalities are related to severe neurological disorders, including aseptic meningitis, cerebellar encephalitis, and acute flaccid paralysis [4],[7]. EV71 has been described as the second most important neurotropic virus after poliovirus [8]. In fatal cases, neuronal degeneration is evident and EV71 can be isolated from regions of the central nervous system (CNS), including spinal cord, medulla oblongata, and pons. Encephalitis and CNS damage during EV71 infection is likely due to the neurotropic characteristics of the virus [9],[10]. Several reports showed that human endothelial and neurons are targets of EV71 infection, and apoptosis has been described in infected cells [11]–[13]. CNS infection by EV71 has also been reported in animal models, including mice, and cynomolgus and rhesus monkeys [14]–[18]. In order to develop effective vaccines and antiviral therapies against EV71, it is important to understand the pathogenesis of EV71 infection.

Autophagy is a biological process involving the degradation of aggregated proteins and damaged organelles to maintain homeostasis [19]. Aberrant autophagy may lead to various pathogenic conditions, including diabetes, neuron degeneration, heart disease, and cancers [20],[21].

Autophagic flux involves the formation of phagophores, autophagosomes, and autolysosomes, as well as degradative processes in the vesicles [19]. During autophagic progression, the phagophore is initiated followed by nucleation and elongation, leading to the formation of a double-membrane vesicle, which is designated an autophagosome. After recruitment of aggregated proteins and damaged organelles, the autophagosome then fuses with the lysosome to form the autolysosome. Alternatively, the autophagosome may fuse with the endosome to form a vesicle known as an amphisome [22],[23]. Finally, the sequestered proteins or organelles are digested by proteases for recycling [21],[24]. This process prevents cell death under conditions of nutrient deprivation, growth factor depletion, and other stresses.

Accumulated evidence indicates that pathogen infection (including bacterial, viral, and parasitic infection) induces autophagy [21],[25]. Furthermore, certain viruses, such as HSV-1, Kaposi’s sarcoma-associated herpesvirus, and murine γ-herpesvirus 68, have evolved mechanisms to evade the host autophagic response [26]–[28]. In contrast, other viruses, such as poliovirus, rhinovirus, coronavirus, Epstein-Barr virus, dengue virus, hepatitis C virus, HIV, coxsackievirus B3, and EV71, induce autophagic activity [29]–[36]. Virus-mediated autophagy may enhance viral replication or evade immune surveillance [37]. We previously reported that EV71 infection can induce autophagic machinery to enhance viral replication *in vitro*[36]. Wang *et al.* developed a mouse model which mimics the natural route of EV71 infection in humans. Mice can be infected orally by mouse-adapted EV71 (MP4 strain), which infects CNS neurons [16]. Using Wang *et al.*’s adapted virus (EV71 MP4) and the mouse model, we further demonstrated that this virus can induce autophagy in the brain tissues of the infected mice [36]. We also reported that dengue virus (DV) serotype-2 infection of suckling mice induces autophagy, which plays a promoting role in DV replication and pathogenesis [38]. However, these previous reports did not clarify whether EV71 infection can induce an autophagic flux and did not show the effects of EV71-induced autophagy on physiopathological responses and viral titers in the infected mice. Therefore, in the present study, the same mouse model was utilized to clarify the pathological effects of induced autophagy *in vivo* during EV71 infection.

## Methods

### Cell line and virus

Human neuroblastoma (SK-N-SH, ATCC: HTB-11) and human rhabdomyosarcoma (RD, ATCC: CCL-136) cells were grown in L-glutamine containing Dulbecco’s modified Eagle’s medium (DMEM) and in Eagle’s modified essential medium (EMEM) (GIBCO-BRL, Grand Island, NY, USA) supplemented with 10% FBS (Trace BioSciences, Sydney, Australia), 1% sodium pyruvate (GIBCO), plus penicillin-streptomycin (200 unit/ml) at 37°C in a 5% CO_2_ incubator. The EV71 strain 4643 was isolated from a patient in Taiwan and the mouse-adapted strain MP4 was kindly provided by Dr. Chun-Keung Yu, National Cheng Kung University, Tainan, Taiwan. Viruses were generated and titrated in RD cells by plaque assay and stored at −80°C [36]. EV71 inactivation (iEV) was conducted by exposing the virus to UV (wavelength 225 nm) for 30 min. Viral viability was confirmed by plaque assay.

### Immunohistochemical and immunofluorescence staining

SK-N-SH cells (2 × 10^5^ cells/well) were seeded onto a 6-well plate (TPP, Trasadingen, Switzerland) and incubated at 37°C overnight. After virus infection at indicated times, the percentage of cells showing the LC3 punctate aggregation was counted under a fluorescence microscope (Olympus FB1000, Tokyo, Japan). Cells containing ≧5 punctate GFP-LC3 localization were defined as autophagy-positive cells. Thus, the percentage of cells showing significant punctate formation was considered to be the number of autophagy-positive cells relative to GFP-expressing cells. EV71 antigens (structural protein VP1 and non-structural protein 2C), autophagy protein LC3, and late endosome protein mannose 6-phosphate receptor (MPR) expression in EV71-infected cells were detected by indirect immunofluorescence labeling. At various times post-infection, cells were washed twice with PBS, then fixed with 3.7% paraformaldehyde in PBS for 15 min. After rinsing with PBS three times per 5 min, the cells were subsequently permeabilized with 0.1% Triton X-100 in PBS for 15 min, then washed three times with PBS. After washing, cells were immersed with SuperBlock® Blocking Buffer in PBS (Thermo Scientific, Rockford, IL, USA) for 1 hr at RT, then incubated with one or two primary antibodies at 4°C overnight. Following incubation, cells were washed with PBS six times per 10 min, then incubated with appropriate secondary antibodies for 1 hr at RT. Subsequently, the samples were washed with PBS six times per 10 min and mounted with VECTASHIELD Mounting Medium® (Vector Labs, Burlingame, CA, USA) onto glass slides. Finally, the samples were investigated under a confocal microscope (Olympus FluoView FV1000, Tokyo, Japan). The primary antibodies used were a 1:50 dilution for rabbit polyclonal anti-MAP-LC3 antibody (Abgent, Flanders Court, San Diego, CA, USA), a 1:50 dilution for mouse monoclonal anti-MAP-LC3 antibody (Abgent), a 1:150 dilution for rabbit polyclonal anti-Mannose 6 Phosphate Receptor antibody (Abcam, Cambridge, MA, USA), a 1:50 dilution for mouse monoclonal anti-EV71 VP1 antibody (Chemicon, Temecula, CA, USA), and a 1:50 dilution for rat polyclonal anti- EV71 2C antibody (a gift from Dr. Jim-Tong Horng) [39]. The secondary antibodies used were a 1:200 dilution for Alexa Fluor® 488 goat anti-mouse IgG (Invitrogen, Carlsbad, CA, USA), Alexa Fluor® 488 goat anti-rabbit IgG (Invitrogen), Alexa Fluor® 594 goat anti-mouse IgG (Invitrogen), Alexa Fluor® 594 goat anti-rabbit IgG (Invitrogen), and Alexa Fluor® 594 goat anti-rat IgG (Invitrogen).

### Western blot analysis

Cells in the plate were washed with PBS and then incubated with 80 μl of modified RIPA lysis buffer (1 ml of lysis buffer was prepared by mixing 1 ml of RIPA solution, 10 μl of PMSF (0.1 M), 10 μl of aprotinin (2 mg/ml), 20 μl of EGTA (0.1 M), 5 μl of EDTA (0.1 M), 5 μl of leupeptin (2 mg/ml), and 4 μl of sodium orthovanadate (Na_3_VO_4_, 0.5 M) per 10-cm cell culture dish. Cell lysates were harvested by scraping, followed by centrifugation at 14,000 rpm at 4°C for 20 min, and then stored at −70°C. The supernatants were normalized for equal protein content (BCA assay, Pierce, Rockford, IL, USA). Equal amounts of protein were subjected to sodium dodecyl sulfate-polyacrylamide gel electrophoresis (SDS-PAGE). Proteins in the gel were transferred to the PVDF membrane (Millipore, Billerica, MA, USA) and subsequently incubated at RT with 5% non-fat dried milk in TBST wash buffer for 1 hr. After rinsing with TBST, the membranes were then incubated overnight at 4°C with specific primary antibodies in TBST. Following incubation, the membranes were washed with TBST three times for 30 min and incubated with a 1:5000 dilution of anti-rabbit (Amersham Pharmacia, Piscataway, NJ, USA) or anti-mouse (Chemicon, Temecula, CA, USA) IgG antibody conjugated with horseradish peroxidase at RT for 1 hr. After incubation with enhanced chemiluminescence (ECL) solution (Millipore, Billerica, MA, USA) for 1 min, the membrane was exposed to an X-ray film (Eastman Kodak, NY, USA). The Western blotting results were quantified by densitometric analysis using VisionWorks™ LS image acquisition and analysis software (UVP, Upland, CA, USA).

### Plaque assay

RD cells (2 × 10^5^ cells/well) were plated onto a 24-well plate (TPP) and incubated at 37°C for 16–20 hr. When the complete medium was removed, cells were infected with serial diluents (100 μl/well) of the virus at 10-fold concentrations. The serial viral suspension was diluted in DMEM medium containing 2% FBS. After absorption at 37°C and shaking every 15 min for 1 hr, the viral suspension was replaced with 2-fold DMEM containing 2% FBS and 1% methyl cellulose solution (American Biorganics, Niagara Falls, NY, USA). The medium was discarded at day 3 p.i. The cells were washed with PBS, then fixed and stained with 10% crystal violet at 37°C for 1 hr. Finally, the crystal violet was rinsed off with distilled water and dried by heat. Plaque-forming unit per milliliter (pfu/ml) was used to represent the viral titer.

### Virus inoculation of the ICR suckling mice

Seven-day-old ICR mice (purchased from Laboratory Animal Center, National Cheng Kung University, College of Medicine, Tainan, Taiwan), were intracranially inoculated with mouse-adapted strain EV71 MP4 (5 × 10^5^ pfu/mouse). Control mice were inoculated with DMEM medium containing 2% FBS. Mice were monitored daily for 6 to 10 days to measure body weight, evaluate clinical signs, and record mortality. Clinical symptoms were scored as follows: 0: healthy; 1: ruffled hair, hunchbacked appearance or reduced mobility; 2: wasting; 3: forelimb or hindlimb weakness; 4: forelimb or hindlimb paralysis; and 5: moribund or death. The mice experiment protocols were approved by the Laboratory Animal Committee at National Cheng Kung University. The mice were maintained at the Animal Facility of National Cheng Kung University and were manipulated according to the animal experiment guidelines of the National Science Council, Taiwan.

### Statistical analysis

The body weight and clinical scores of the mice, and the viral titer in this study, were analyzed by the Mann–Whitney U test, and the survival rates of the mice were analyzed by log rank analysis. Data are presented as the mean ± standard deviation. Differences between the test and control groups were analyzed by the Student’s t test using the Prism software. A p value of <0.05 was considered significant.

## Results

### EV71 infection of human neuroblastoma SK-N-SH cells induced amphisome formation and autophagic flux

**Figure 1:**
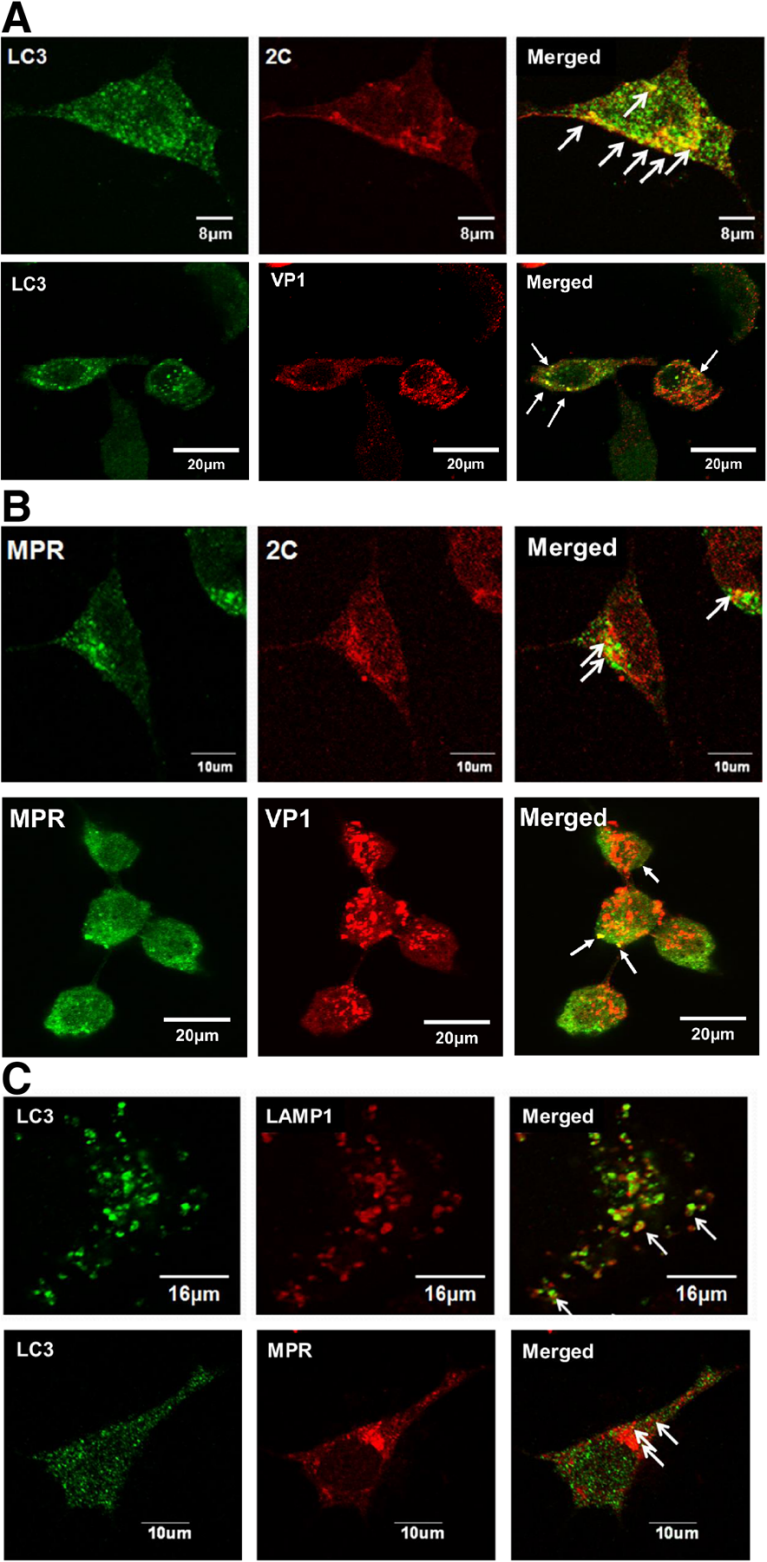
**The formation of autophagosome and amphisome accompanied with EV71 structural protein VP1 and nonstructural protein 2C was detected in EV71 infected SK-N-SH cells.** SK-N-SH cells were infected with EV71 (MOI = 10) for 9 hr. **(A)** The cells were treated with anti-LC3 rabbit polyclonal antibody and either anti-EV71 2C rat polyclonal antibody or anti-EV71 VP1 mouse monoclonal antibody, then incubated overnight at 4°C and investigated under a confocal microscope. Green: LC3; Red: EV71 2C and VP1; Yellow: colocalization of LC3 and EV71 2C or VP1. **(B)** SK-N-SH cells were treated with MPR rabbit polyclonal antibody and either EV71 2C rat polyclonal antibody or VP1 mouse monoclonal antibody. Green: MPR; Red: EV71 2C and VP1; Yellow: colocalization of MPR and EV71 2C or VP1. **(C)** The cells with or without EV71 infection were then treated with LC3 mouse monoclonal antibody and LAMP1 mouse monoclonal antibody or mannose-6-phosphate receptor (MPR) rabbit polyclonal antibody. Green: LC3; Red: LAMP1 and MPR; Yellow: colocalization of LC3 and LAMP1 or MPR. Arrow indicates colocalization.

We previously reported that EV71 infection can induce autophagy activity, which further promotes viral replication, in human rhabdomyosarcoma RD and neuronal SK-N-SH cells [36]. This study further investigated whether viral structure protein VP1 and nonstructural protein 2C may colocalize with the double-membrane autophagosome and endosome using anti-VP1 and anti-2C antibody, respectively. We also determined whether amphisome and autophagic flux are induced in EV71-infected cells. Our data showed that in EV71-infected cells, LC3 puncta (Green, a marker of autophagosome) abundantly colocalized with the EV71 structural protein VP1 and nonstructural protein 2C under confocal microscopy (Figure 1A, arrow), suggesting that the EV71 VP1 and 2C proteins are distributed around autophagosomes. We further revealed that the EV71 VP1 and 2C proteins colocalized with the MPR protein (Mannose-6-phosphate receptor, a marker of late endosome) (Figure 1B, arrow), which parallel with the increased colocalization of the LC3 and MPR proteins in EV71-infected cells (Figure 1C, arrow). At the same time, we also detected colocalization of LC3 and LAMP1 (lysosome marker) in EV71-infected cells, indicating that EV71 infection induces autolysosome formation (Figure 1C, arrow). Our data reveal that the endosome with EV71 fuses with the autophagosome to form the amphisome, which is consistent with the results of a study by Khakpoor *et al.* on DV infection [23]. In summary, EV71 infection can induce autophagosome, amphisome and autolysome formation, and the structural protein VP1 and nonstructural protein 2C of EV71 were distributed around the autophagosome and amphisome.

**Figure 2:**
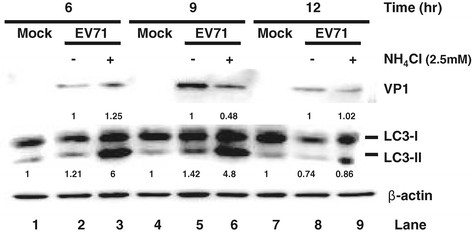
**Autophagic flux was induced in EV71-infected SK-N-SH cells.** SK-N-SH cells were infected with EV71 for various times at MOI of 10. Cells infected with EV71 were treated with or without ammonium chloride NH_4_Cl (2.5 mM) for 1 hr, and the expression levels of EV71 VP1 and LC3-II were evaluated by Western blotting using specific antibodies. β-actin was used as the internal control. The numbers under each band represent the intensity of each band measured by densitometric analysis using VisionWorks^TM^ LS image acquisition and analysis software (UVP, Upland, CA, USA). For the comparison of LC3-II levels, we set the intensity of Mock at each time point as 1 (normalized with the intensity of β-actin). For the comparison of VP1 levels, we set the intensity of EV71 infection without NH4Cl as 1 at each time point (normalized with the intensity of β-actin).

To determine whether EV71 infection induces autophagic flux, the time course of autophagic progression was investigated. Our data showed that the LC3-II expression level was gradually increased and reached the peak at 9 hr post-infection (p.i.). The expression of LC3-II was then decreased after 12 hr p.i. (Figure 2, lane 5 and lane 8), indicating the progression of autophagy. To further confirm that EV71 can induce autophagic flux, the degradation of LC3-II expression was blocked by NH_4_Cl, which exerts its effect by neutralizing the acidic pH and blocking lysozyme degradation. Our data showed that in the presence of NH_4_Cl at 6 hr, 9 hr, and 12 hr p.i., the expression level of LC3-II was increased as compared to the levels in the EV71-infected group without NH_4_Cl treatment and the mock control groups (Figure 2, lane 3, lane 6, and lane 9). Autophagic flux was further confirmed using treatment with vinblastine, a microtubule depolymerizing agent that causes the accumulation of autophagic vacuoles by preventing their degradation (Additional file 1). The above data indicate that EV71 infection can induce an autophagic flux which can be blocked by autophagy blockers NH_4_Cl and vinblastine. Altogether, EV71 infection can induce an autophagic flux including autophagosome, amphisome and autolysome formation. The structural protein VP1 and nonstructural protein 2C of EV71 were distributed around the autophagosome and amphisome. However, the roles of these viral proteins in autophagy progression remain to be determined.

### EV71 infection of the ICR suckling mice caused physiopathological changes and mortality

**Figure 3:**
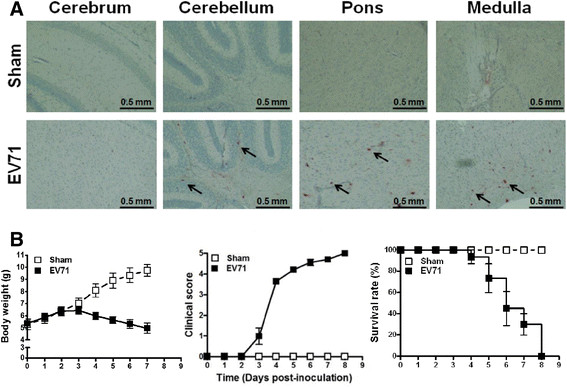
**EV71 infection caused physiopathological changes and mortality of the suckling mice. (A)** Seven-day-old ICR suckling mice were inoculated with the EV71 mouse-adapted strain MP4 (5 × 10^5^ PFU/mouse). The expression of EV71 VP1 in the brain tissues of control and EV71-infected suckling mice at day 2 p.i. was detected by immunohistochemical staining using anti-VP1 antibody. Control (sham) group was given DMEM medium containing 2% FBS. Arrow indicates location of EV71 VP1. **(B)** Seven-day-old ICR suckling mice (n = 6, each group) were intracranially inoculated with the mouse-adapted strain of EV71, MP4 (5 × 10^5^ pfu/mouse). The body weight, clinical symptoms, and survival rate were monitored daily after inoculation. Clinical score was defined as follows: 0: Healthy, 1: Ruffled hair/Hunchback appearance/Reduced mobility, 2: Wasting, 3: Forelimb or hindlimb weakness, 4: Forelimb or hindlimb paralysis, 5: Death. Values are the means ± standard deviation of the results of three independent experiments.

We previously reported that dengue virus type 2 and mouse-adapted EV71 (MP4 strain) infection induce autophagy in the brain tissues of ICR mice [36],[38]. To further clarify the effect of EV71 infection-induced autophagy on pathogenesis as well as viral titer, 7-day-old ICR mice were used. After intracranial inoculation with EV71 (5 × 10^5^ pfu/mouse), mice were monitored daily to assess body weight, disease symptoms, and survival rate. Initially, we confirmed by immunohistochemical staining that in the brains of EV71 MP4-infected mice, the structural protein VP1 was detected within the cerebellum, pons and medulla (Figure 3A, arrow) of the infected brain comparing to the uninfected control (sham). VP1 antigen was undetectable in cerebrum of EV71 infected brain (Figure 3A). The body weight of the uninfected mice (sham) was steadily increased whereas EV71-infected mice transiently gained weight until day 3 after viral challenge followed by weight loss (Figure 3B, left panel) and death (Figure 3B, right panels). Moreover, the clinical scores were significantly increased from day 3 in EV71-infected mice (Figure 3B, middle panel). These effects were further demonstrated to be virus dose-dependent (Additional file 2), indicating that EV71 infection of the suckling ICR mice influenced body weight, disease symptoms, and survival. Altogether, the above data demonstrate that EV71 infection of ICR mice causes body weight loss, increased disease symptoms and a higher mortality rate.

### EV71 infection induced amphisome and autophagosome formation as well as autophagic flux in the brain tissues of infected mice

**Figure 4:**
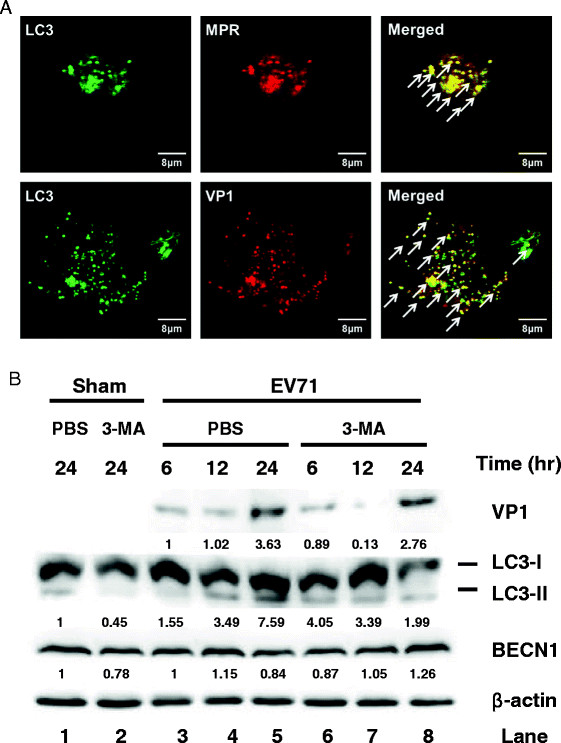
**Autophagy accompanied by autophagosome and amphisome formation was induced in the brain tissues of EV71 MP4-infected suckling mice.** Seven-day-old ICR suckling mice were inoculated intracranially with EV71 MP4 (5 × 10^5^ pfu/mouse). Mice were sacrificed at 24 hr p.i.. **(A)** Autophagosome and amphisome formation was detected in the brain stem of the EV71 MP4-infected suckling mice. The tissue sections were treated with either mouse monoclonal or rabbit polyclonal anti-LC3 antibody and anti-MPR rabbit polyclonal antibody anti-EV71 VP1 mouse monoclonal antibody. Green: MPR; Red: EV71 VP1; Yellow: colocalization of MPR and EV71 VP1. Arrow indicates colocalization. **(B)** Seven-day-old ICR suckling mice (n = 3, each group) were pretreated with 3-MA (0.3 mg/mouse) 2 hr before inoculation of EV71 MP4 (5 × 10^5^ pfu/mouse). Mice brain tissues were harvested and total protein lysate was collected after EV71 infection for 6 hr, 12 hr and 24 hr. The total protein lysate from each group was pooled together and the expression levels of EV71 VP1, LC3-II, and BECN1 were measured by Western blotting using specific antibodies. β-actin was used as the internal control. The numbers under each band are the quantification of the band intensity. For the comparison of VP1 protein expression levels, the intensity of EV71 infected cells without 3-MA treatment at 6 hr p.i. was set as 1 (normalized with the intensity of β-actin). For the comparison of LC3-II and BECN1 levels, the intensity of Sham PBS control cells without 3-MA treatment at 24 hr p.i. was set as 1 (normalized with the intensity of β-actin).

The formation of autophagosome-like vesicles in the neurons of infected mice indicates that EV71 infection induces autophagy *in vivo*[36]. To further confirm that EV71 infection indeed induces the formation of autophagosome and amphisome as well as autophagic flux in mice, sections of brain tissues of the infected mice were collected and the ultrastructure of the vesicles in the infected brain tissues were investigated by immunofluorescence staining under confocal microscopy. The results showed that LC3 puncta, which represent autophagosomes, were colocalized with EV71 VP1 protein in the brain tissues of EV71-infected mice at 24 hr p.i., suggesting that EV71 infection can induce autophagosome formation (Additional file 3 and Figure 4A lower panel). This finding is consistent with the results of our previous TEM investigation that showed VP1 protein colocalized with autophagosome-like vesicles [36]. Moreover, EV71 infection-induced LC3 puncta were also colocalized with MPR protein (representing endosome) (Figure 4A upper panels, arrow), indicating that endosome may fuse with the autophagosome to form amphisome *in vivo* during EV71 infection, which is consistent with the result of the *in vitro* study (Figure 1C, lower panel arrow). We further investigated EV71 infection-induced LC3-II and VP1 expression in the brain tissues of the infected mice at 6 hr, 12 hr and 24 hr p.i. by Western blotting. Figure 4B showed that VP1 expression was detected and increased in the brains of the mice from 6 hr to 24 hr p.i. (Figure 4B, lanes 3, 4 and 5). Accordingly, increased LC3-II expression was also detected from 6 hr to 24 hr p.i. (Figure 4B, lanes 3, 4 and 5) compared to the uninfected sham control (Figure 4B, lane 1). Autophagy inhibitor 3-MA was used to block autophagic activity during EV71 infection to further confirm the effect of EV71-induced autophagy on viral production. Figure 4B showed that LC3-II expression was suppressed 55% by 3-MA in Sham control mice without infection (Figure 4B, lane 2), and EV71-induced LC3-II expression was suppressed about 73% accompanied with VP1 level was suppressed about 24% at 24 hr p.i. in the presence of 3-MA compared with that of the EV71-infected group without 3-MA treatment (Figure 4B, lane 8 vs. lane 5). Above results suggest that both endogenous as well as EV71-induced autophagy could be blocked by 3-MA, EV-71-induced autophagy affects viral production in the infected mice brains. In our previous report, manipulation of autophagy with 3-MA, rapamycin, tamoxifen and starvation can affect EV71 titer*,* suggesting that autophagy plays a supportive role in EV71 replication, and the inhibitor 3-MA showed no side effect both *in vitro* and *in vivo*[38]. The result that 3-MA only partially suppressed EV-71 VP1 expression compared to LC3-II expression at 24 h p.i. is consistent with our published reports of dengue virus [38] and EV71 [36] that EV71 and dengue virus-induced autophagy only plays a supportive role in viral replication. Therefore, suppressing autophagy virus can still replicate but to a less amount. In addition, the expression level of Beclin-1 (BECN1) showed no significant change, indicating that it is not involved in EV71-induced autophagy. A similar result was seen in dengue virus-infected suckling mice [38]. In summary, EV71 infection of the suckling mice can induce an autophagic flux, which involves autophagosome and amphisome formation in the brain tissues.

### EV71 infection-induced autophagy in mice increased disease severity and viral titer

**Figure 5:**
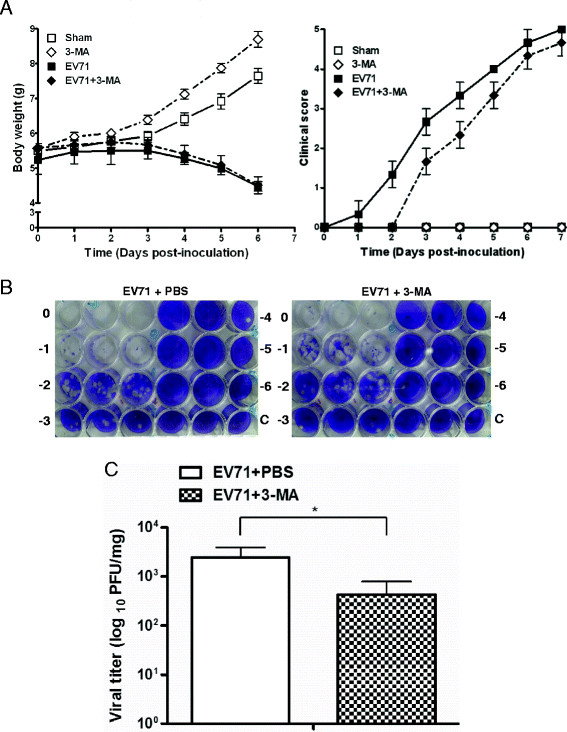
**Suppression of EV71 MP4-induced autophagy in mice reduced EV71-related pathogenesis and viral replication.** Seven-day-old ICR suckling mice (n = 3, each group) were inoculated intracranially with 3-MA (0.3 mg/mouse) 2 hr before inoculation with EV71 MP4 (5 × 10^5^ pfu/mouse). **(A)** The body weight and disease symptoms were monitored daily after inoculation for 6 and 7 days, respectively. **(B)** The mice received the same treatment as in **(A)**, and the brain tissues of the mice were weighed and immersed in DMEM medium containing 2% FBS (with equal volume) at 12 hr p.i., and viral titer was determined by plaque assay. The viral titer was calculated based on the weight of the mice brain. Results are the mean ± stand deviation of each group. PBS treatment was used as the control. **(C)** Quantitative presentation showed the viral titer detected in infected mice brains with or without 3-MA. Values are the means ± standard deviation of results of three independent experiments. Student’s *t* test was used *P < 0.05 (compared to the value of EV71 + PBS).

We previously reported that the MP4 strain of EV71 can induce autophagy in suckling mice [36]. However, the effects of EV71-induced autophagy on disease symptoms, mortality, and viral replication *in vivo* remain undetermined. To evaluate the pathological effect of EV71-induced autophagy on the infected mice, autophagy activity was suppressed by 3-MA, and the physiopathological parameters, including the body weight and disease symptoms of the mice, were monitored daily after EV71 infection for 6 and 7 days, respectively. Our data showed that the body weight of the infected mice, both with and without 3-MA treatment, significantly dropped from day 3 to day 6 (Figure 5A, left panel), indicating that autophagy played no specific role in the loss of body weight of the infected mice. Furthermore, the disease symptoms were detected from day 1 to day 7 p.i. in the EV71-infected mice. The detection of disease symptoms was delayed to day 3 p.i. in the EV71-infected mice with 3-MA (EV71 + 3-MA), whereas the uninfected (sham) and 3-MA-only controls showed no disease symptoms at all (Figure 5A, right panel). To determine the viral titer, the brains of the infected mice shown in Figure 5A were collected and subjected to plaque assay. Our data showed that the titer of EV71 in the 3-MA treated group was significantly reduced compared with that of the PBS-treated group at 12 p.i. (Figure 5B and 5C), indicating that EV71-induced autophagy promotes viral replication *in vivo,* which was similar to the result of our *in vitro* investigation [36]. Taken together, our findings showed that EV71 infection-induced autophagy increased viral titer and disease severity in the suckling mice.

## Discussion

We have reported that EV71 infection of human rhabdomyosarcoma RD and neuronal SK-N-SH cells both induce autophagy, which is beneficial for viral replication. Our investigation of the signaling pathway revealed that the decreased expression of phosphorylated mTOR and phosphorylated p70S6K is involved in EV71-induced autophagy in a cell-specific manner. Other signaling pathway molecules, including extracellular signal-regulated kinase (Erk), PI3K/Akt, Bcl-2, BNIP3, and Beclin-1, are not involved. Electron microscopy showed colocalization of double-membrane autophagosome-like vesicles with EV71-VP1 and LC3 protein in the brain tissue of the ICR mice infected by EV71. These data indicate that EV71 infection triggered autophagic activity and induced autophagosome-like formation both *in vitro* and *in vivo*[36]. However, this report did not clarify whether EV71 infection can induce an autophagic flux and did not show the effect of EV71-induced autophagy on the physiopathological responses and viral titer of the infected mice. Here, we demonstrated that EV71-induced autophagy could indeed trigger the autophagic flux and amphisome formation in human neuronal SK-N-SH cells (Figures 1 and 2 and Additional file 1). Panyasrivanit *et al.* have shown that the DV2 titer is increased by blocking autophagic flux using fusion blocker L-asparagine, suggesting that the autophagic flux process decreases DV2 titer [22]. However, blocking autophagic flux by NH_4_Cl or vinblastine (Vin) decreased VP1 expression, indicating that autophagic flux is beneficial for EV71 replication. While these findings shed light on the role of the autophagic flux in EV71 replication, further clarification is needed.

Overexpression of the hepatitis B virus X gene enhances starvation-induced autophagy through the upregulation of Beclin 1 expression [40]. Poliovirus 2BC and 3A proteins regulate LC3 modification and membrane induction [29],[41]. DV2 NS4A protein induces LC3 cleavage and translocation in epithelial cells [42]. Furthermore, we compared viral structural protein VP1 and nonstructural protein 2C for their relationship with autophagosome and amphisome and found that these two proteins were abundantly colocalized with LC3 protein around the autophagosome (Figure 1A) in the infected cells. Tang *et al.* reported that the EV71 2C protein is associated with host membrane vesicles, which form viral replication complexes where viral RNA synthesis takes place [39]. Greninger *et al.* reported that enteroviruses utilize their 3A and 2BC proteins to reorganize cellular membranes associated with the Golgi apparatus [43]. It is possible that EV71 nonstructural 2C protein may form a replication complex with RNA on the autophagosome. A similar phenomenon has been reported with other viral infections [32],[34],[44]–[46]. Poliovirus and Coxsackieviruses B3 and B4 may induce the accumulation of membrane-like structures in the host cytoplasm early after infection [29],[34],[47]. In addition, The RNA replication complex of DV2, DV3, HIV-1, and influenza A virus, including viral RNA, structural, and nonstructural proteins, were colocalized with the autophagosomes or autolysosomes in the infected cells [22],[32],[48],[49]. Other reports showed that autophagic activation during virus infection, including influenza A virus, ectromelia virus, chikungunya virus, and flavivirus, can delay or block cell death to enhance viral replication [42],[50]–[52]. Xi *et al.* reported that EV71 infection of the rhabdomyosarcoma RD-A cells can induce both autophagy and apoptosis, and inhibition of autophagy may either inhibit or enhance apoptosis depending on the time of inhibition. Furthermore, inhibition of apoptosis induces autophagy [53]. Therefore, whether the autophagosome and amphisome complex are the sites for EV71 replication warrants further investigation. Additionally, further research is needed to establish whether EV71-induced autophagy enhances EV71 replication by suppressing apoptosis.

The endocytotic pathway requires the acidification of endosomes to induce the fusogenic activity of the viral fusion proteins, and this phenomenon has been reported to facilitate the entry of the virus during viral replication [22],[54],[55]. In this study, we detected the presence of EV71 protein VP1 and 2C in the endosome, which further fuses with the lysosome to form the amphisome in the infected cell at 24 hr p.i. (Figure1A, 1B and 1C). A similar phenomenon was reported in dengue virus-infected cells [22]. Because of the limitation of antibodies, our *in vivo* study only detected colocalization of the LC3 protein with the EV71 VP1 and MPR proteins (endosome markers) (Figure 4A, lower panel). Nevertheless, this indicates that EV71 infection could also induce amphisome formation in the brain tissues of the mice.

We further revealed that EV71 increased autophagic activity in the brain tissues of the infected mice (Figure 4B), which was associated with the increase of disease symptoms and elevation of virus titer (Figure 5A and 5B).

We also showed that in the infected suckling mice, the disease severity was attenuated at the early stage (from day 2 to day 4 p.i.) after 3-MA treatment. Furthermore, the results revealed that the viral titer was significantly suppressed in the brain stem of the infected suckling mice after 3-MA treatment. This indicates that autophagy is involved in EV71-related pathogenesis and promotes viral replication *in vivo*. These results are similar to the findings of our previous report that showed DV2 infection of the suckling mice could also induce autophagy, which plays a promoting role in DV replication and pathogenesis [38]. Based on above results, we hypothesize that autophagy increases EV71 infection-related pathogenesis of the mice, at least partially, through the promotion of viral replication. However, blockage of autophagy by 3-MA showed no significant increase of the survival rate of EV71-infected mice (data not shown), which was similar to the result of the DV2-infected mice [38]. Wu *et al.* reported that 3-MA plays dual roles in autophagy. Under starvation conditions, 3-MA suppresses PI3K class III and inhibits autophagy; in contrast, under normal conditions, 3-MA promotes autophagic flux [56]. Therefore, the treatment conditions of 3-MA need to be further optimized to increase its inhibitory effect on autophagy. Further research using autophagy gene knockout mice is needed to obtain conclusive *in vivo* evidence of the involvement of autophagy in the EV71-related pathogenesis*.*

The possible reason that 3-MA treatment significantly reduced viral yields at 12 h; however, such treatment only resulted in a delayed onset of clinical illness by 2 days, compared to PBS-treated controls, without significantly affecting weight loss is the short half-life and instability of 3-MA *in vivo*.

## Conclusion

Our study demonstrated that EV71 infection induces the autophagic flux including the formation of amphisome and autophagosome in the brain tissues of the suckling mice. EV71-induced autophagy promotes viral replication and increases the severity of pathogenesis both *in vitro* and *in vivo*. Therefore, the role of autophagy regulation in EV71-infected patients warrants further investigation.

## Competing interests

The authors declare that they have no competing interests.

## Authors’ contributions

YRL conducted this project and wrote the manuscript. PSW executed the experiments. JRW conceived the plan. HSL initiated this project and proposed the fundamental frame of this project. All authors read and approved the final manuscript.

## Additional files

## Supplementary Material

Additional file 1: **EV71-induced autophagic flux was confirmed by blocking the fusion of autophagosome and lysosome in SK-N-SH cells during EV71 infection.** SK-N-SH cells were infected with EV71 in the presence or absence of vinblastine (Vin, 20 μM) treatment. The expression levels of EV71 VP1 and LC3-II were determined by Western blotting using specific antibodies. β-actin was used as the internal control. For the comparison of LC3-II levels, we set the intensity of Mock at 6 hr p.i. as 1 (normalized with the intensity of β-actin). Click here for file

Additional file 2: **The clinical score and mortality rate of the infected mice were affected in an EV71 dose-dependent manner.** Seven-day-old ICR suckling mice (n = 4-6, each group) were inoculated intracranially with different doses of EV71 MP4, which resulted in a dose-dependent effect on clinical scores and mortality of the infected mice. β-actin was used as the internal control. The numbers under each band are the quantification of the band intensity compared to the mock control. Click here for file

Additional file 3: **Autophagosome formation was detected in the brain tissues of EV71 MP4-infected suckling mice.** Seven-day-old ICR suckling mice were inoculated with the EV71 mouse-adapted strain MP4 (5 × 10^5^ pfu/mouse). Mice were sacrificed at 24 hr p.i.. The tissue sections were treated with anti-LC3 rabbit polyclonal antibody and anti-EV71 VP1 mouse monoclonal antibody and incubated overnight at 4°C. Autophagosome formation was then investigated under a fluorescence microscope. Green: LC3; Red: EV71 VP1; Yellow: colocalization of LC3 and EV71 VP1 (arrow). Click here for file
