# Supplementary figures and images for: Enterovirus 71-induced autophagy increases viral replication and pathogenesis in a suckling mouse model
Source: J Biomed Sci. 2014 Aug 20;21(1):80. doi: 10.1186/s12929-014-0080-4 (PMC4237791; doi:10.1186/s12929-014-0080-4)

## Slide 1
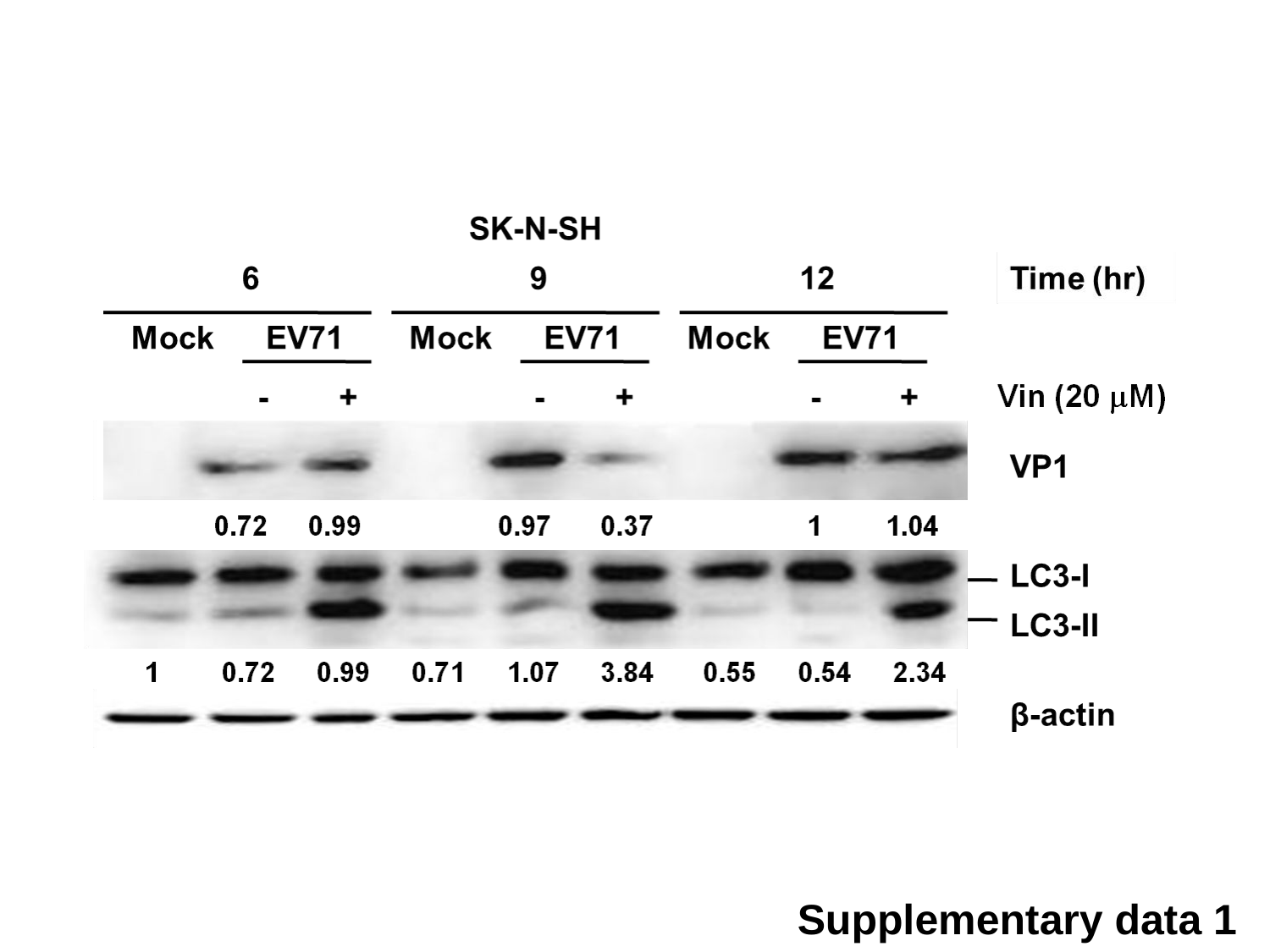

Supplementary data 1

Supplement: Additional file 1: — EV71-induced autophagic flux was confirmed by blocking the fusion of autophagosome and lysosome in SK-N-SH cells during EV71 infection. SK-N-SH cells were infected with EV71 in the presence or absence of vinblastine (Vin, 20 μM) treatment. The expression levels of EV71 VP1 and LC3-II were determined by Western blotting using specific antibodies. β-actin was used as the internal control. For the comparison of LC3-II levels, we set the intensity of Mock at 6 hr p.i. as 1 (normalized with the intensity of β-actin). [file s12929-014-0080-4-S1.ppt]

## Slide 1
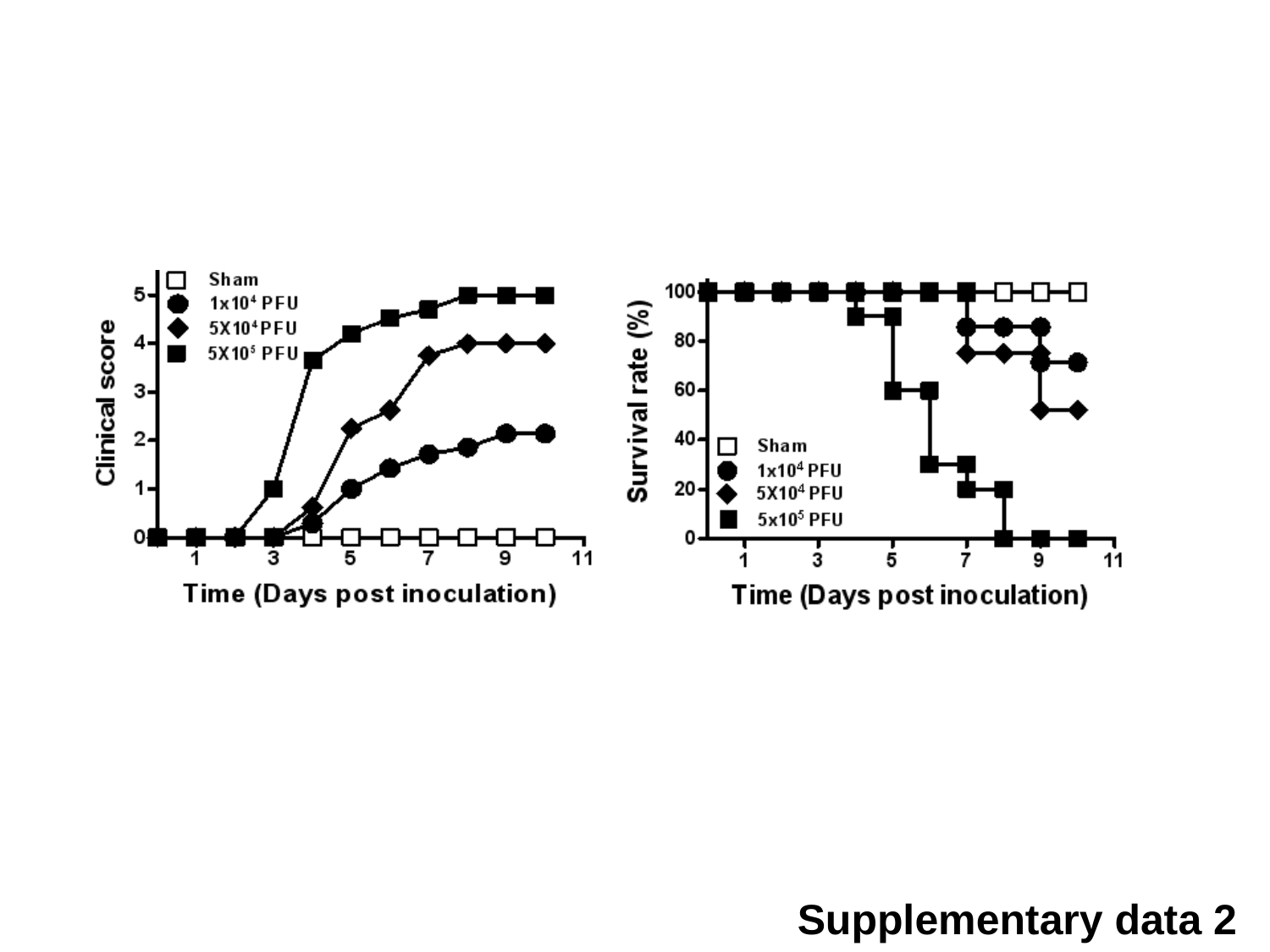

Supplementary data 2

Supplement: Additional file 2: — The clinical score and mortality rate of the infected mice were affected in an EV71 dose-dependent manner. Seven-day-old ICR suckling mice (n = 4-6, each group) were inoculated intracranially with different doses of EV71 MP4, which resulted in a dose-dependent effect on clinical scores and mortality of the infected mice. β-actin was used as the internal control. The numbers under each band are the quantification of the band intensity compared to the mock control. [file s12929-014-0080-4-S2.ppt]

## Slide 1
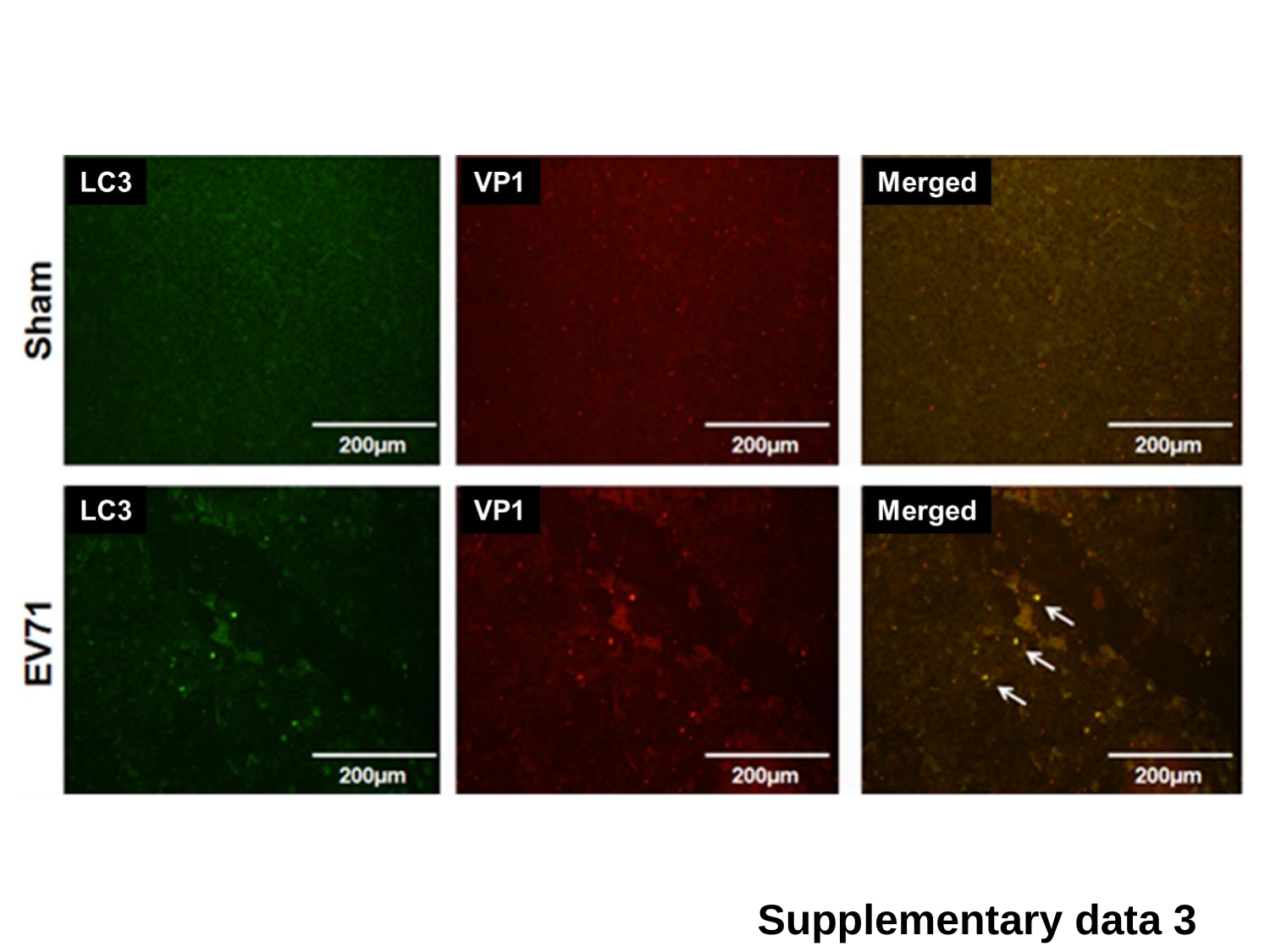

Supplementary data 3

Supplement: Additional file 3: — Autophagosome formation was detected in the brain tissues of EV71 MP4-infected suckling mice. Seven-day-old ICR suckling mice were inoculated with the EV71 mouse-adapted strain MP4 (5 × 10^5^ pfu/mouse). Mice were sacrificed at 24 hr p.i.. The tissue sections were treated with anti-LC3 rabbit polyclonal antibody and anti-EV71 VP1 mouse monoclonal antibody and incubated overnight at 4°C. Autophagosome formation was then investigated under a fluorescence microscope. Green: LC3; Red: EV71 VP1; Yellow: colocalization of LC3 and EV71 VP1 (arrow). [file s12929-014-0080-4-S3.ppt]
